# Preoperative anxiety in patients admitted for brain surgery: A systematic review

**DOI:** 10.1192/j.eurpsy.2021.501

**Published:** 2021-08-13

**Authors:** A. Martinelli, V. Oteri, E. Crivellaro, F. Gigli

**Affiliations:** 1 Medicina E Chirurgia, University of Padova, Padova, Italy; 2 Medicina E Chirurgia, University of Catania, Catania, Italy; 3 Medicina E Chirurgia, University of Milano, Milano, Italy

**Keywords:** preoperative anxiety, brain surgery, quality of life, Systematic review

## Abstract

**Introduction:**

Up to 80% of patients scheduled for surgery experience preoperative anxiety, which may implicate perioperative psychological and physical discomforts. Several studies focused on this phenomenon in neurosurgical setting, still controversial evidence exists.

**Objectives:**

Our aim is to synthesize this evidence, investigating prevalence, implications and therapy of preoperative anxiety in brain surgery patients.

**Methods:**

We performed a systematic review of literature by searching PubMed, Embase, and Cochrane Library databases. Data were extracted using the PICO framework. PRISMA guidelines were applied, and the risk of bias was assessed using the RoB 2 and ROBINS tools, as was the methodological quality of the included studies, following GRADE criteria; we excluded articles with serious risk of bias and/or low quality.

**Results:**

We included 27 articles, accounting for 2558 patients of twelve different countries. Prevalence of anxiety before brain surgery was up to 89%, reaching higher levels in women. Anxiety concerned mostly anesthesia and surgical outcome. No correlation emerged between level of anxiety and laterality, histological type of tumor or survival rate. Before surgery, anxious patients performed worse in cognitive tasks and had worse subjective evaluation of their cognitive abilities. After surgery, preoperative anxiety was associated with depression, longer hospitalization, increase of physical disability and lower quality of life. Effective approaches to reduce anxiety were acupuncture, music therapy, virtual reality and pharmacological support.

**Conclusions:**

Preoperative anxiety in brain surgery patients is a common experience that should not be underestimated to achieve a better perioperative care through early detection and adequate pharmacological or non-pharmacological management.

